# Neobaicalein Inhibits Th17 Cell Differentiation Resulting in Recovery of Th17/Treg Ratio through Blocking STAT3 Signaling Activation

**DOI:** 10.3390/molecules28010018

**Published:** 2022-12-20

**Authors:** Jian-Yu Chen, Ying-Jie Yang, Xue-Qin Ma, Qi Cao, Shan-Shan Wei, Rong-Rong Pan, Li-Hong Nan, Yao-Jun Liu, Yan Cao, Xiao-Yun Tian, Shan Deng, Zai-Xing Cheng, Can-Jian Wang, Tao Chen, Yan-Fang Zheng, Ming-Qing Huang

**Affiliations:** 1 Department of Pharmacology, School of Pharmacy, Fujian University of Traditional Chinese Medicine, No. 1, Hua Tuo Road, Min Hou Shang Jie, Fuzhou 350122, China; 2 Key Laboratory of Hui Ethnic Medicine Modernization, Department of Pharmaceutical Analysis, School of Pharmacy, Ministry of Education, Ningxia Medical University, 1160 Shenli Street, Yinchuan 750004, China; 3 School of Pharmacy, Second Military Medical University, No. 325, Guo He Road, Shanghai 200433, China; 4 Seventh People’s Hospital of Shanghai University of Traditional Chinese Medicine, No. 358, Datong Road, Pudong New Area, Shanghai 200137, China; 5 National Engineering Research Center of Chinese Medicine Solid Preparation Manufacturing Technology, Jiangxi University of Chinese Medicine, Nanchang 330004, China

**Keywords:** neobaicalein (NEO), autoimmune diseases, Th17, signal transducer, activators of transcription 3 (STAT3)

## Abstract

Huangqin is the dried root of *Scutellaria baicalensis* Georgi, which has been widely utilized for heat-clearing (Qingre) and dewetting (Zaoshi), heat-killed (Xiehuo) and detoxifying (Jiedu) in the concept of Traditional Chinese Medicine and is used for treating inflammation and cancer in clinical formulas. Neobaicalein (NEO) is of flavonoid isolated from Huangqin and has been reported to possess prominent anti-inflammatory effects in published work. Th17/Treg balance shift to Th17 cells is an essential reason for autoimmune inflammatory diseases. However, the role NEO plays in Th17 and Treg and the underlying mechanism has not been elucidated yet. Network pharmacology-based study revealed that NEO predominantly regulated IL-17 signaling pathway. Moreover, our result shown that NEO (3–30 μmol/L) down-regulated Th17 differentiation and cellular supernatant and intracellular IL-17A level and tumor necrosis factor α production in a concentration-dependent manner. The further mechanism research revealed that NEO also specifically inhibited phosphorylation of STAT3(Tyr725) and STAT4 (Y693) without influence on activation of STAT5 and STAT6 in splenocytes. Immunofluorescence results illuminated that NEO effectively blocked STAT3 translocated into nucleus. Interestingly, NEO at appreciated dose could only inhibit Th17 cell differentiation and have no effect on Treg differentiation. The present study revealed that NEO effectively inhibited Th17 cell differentiation through specifically blocking the activation of STAT3 signaling without inactivation of STAT5 and STAT6. Additional inhibitory effect on activation of STAT4 by NEO also suggested the potential for antagonism against Th1 differentiation. All work suggested that NEO may be a potential candidate for immunoregulation and treating autoimmune inflammatory diseases through inhibiting immune cell viability and T cell differentiation.

## 1. Introduction

Autoimmune diseases are widespread in clinics all over the world, including rheumatoid arthritis (RA), multiple sclerosis (MS), inflammatory bowel disease (IBD), psoriasis and so on [1,2]. Autoimmune diseases refer to disorders which are characterized by both high self-immune response and intolerance to self-antigen, which result in intensive inflammation and subsequent systemic tissue damage [3]. However, the current therapies fail to meet the requirements for treating RA, MS, and IBD. Therefore, re-understanding autoimmune disease pathogenesis, determining new therapeutic targets and discovering new therapeutic drugs based on these new targets will remain the focus of clinical medicine, biomedical science, and pharmaceutical science research in the future.

Recently, the latest research evidence has provided substantial insight into T helper 17 (Th17) and regulatory T (Treg) cells are essential to monitor autoimmune disease pathological processes [4,5]. The public has traditionally believed that Th17 cells antagonize extracellular bacterial and fungal infections [6]. However, an increasing amount of research has illuminated that Th17 cells promote inflammation by secreting various cytokines (IL-17A/F, TNF-α, IL-1β, and IL-6); afterward, IL-17 activates other neighbor cells and causes their activation; subsequently, neighbor cells produce more pro-inflammatory cytokines, chemokines (IL-8, CXCL1/8/10) and matrix metalloproteinases (MMPs), then accelerates more intensive inflammatory response [7,8,9]. Th17 cells are a subgroup of CD4^+^ T cells and can be specifically recognized by their unique transcription factors, retinoic acid receptor-related orphan nuclear receptor (RORγt) and the expression of IL-17 [10]. Additionally, negative expression of both CXCR3 and CCR6 is also confirmed as markers for successful Th17 differentiation [11]. Additionally, patients with RA or IBD were detected with high levels of Th17 cells and serum IL-17A levels [12]. However, treatment with IL-17 antibodies have been clinically successful for their direct inhibition of IL-17 cytokines [13]. In contrast, Treg cells, another subgroup of CD4^+^ T cells, are characterized by expressing transcription factor Foxp3 and secreting IL-10 or TGF-β to antagonize inflammation response promoted by Th17 cells.

Both Th17 and Treg cells are induced to be differentiated from naïve CD4^+^ T cells (Th0), to decide which one is used dependents on the microenvironment in which the naïve CD4^+^ T cells exist. Signal Transducer and Activator of Transcription 3 (STAT3) is another key transcription factor to facilitate Th17 differentiation through up-regulating RORγt, downstream target genes of STAT3 [14]. Additionally, activation of STAT3 signaling promotes cell proliferation through cell cycle progression and apoptosis inhibition [15]. Therefore, blockage in STAT3 signaling is regarded as effective therapeutic strategy for reversing high self-immune response through inhibiting Th17 differentiation, IL-17A expression, and T cell proliferation. Usually, in the activation of STAT3 signaling, IL-6 binds to IL-6R and recruits gp130 but activation of STAT4 signaling requires IL-12, whereas IL-4 is essential to activate STAT5 and STAT6, which all finally results in phosphorylation of JAKs and subsequent STATs phosphorylation, afterward, dimerization of STATs, translocation into nucleus and initiate transcription process [16].

**Figure 1:**
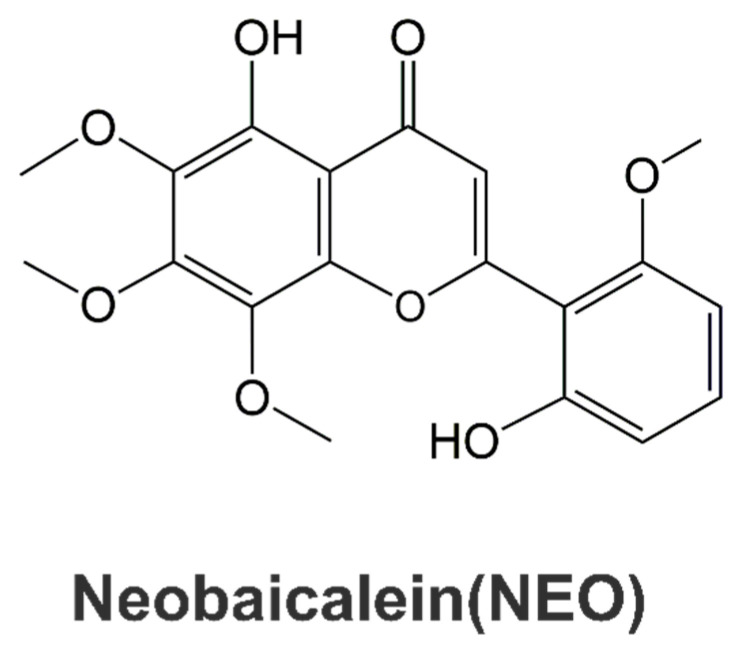
Chemical structural of Neobaicalein (NEO).

Huangqin is the dried root of *Scutellaria baicalensis* Georgi, which has been widely utilized for heat-clearing (Qingre) and dewetting (Zaoshi), heat-killed (Xiehuo) and detoxifying (Jiedu) in the concept of Traditional Chinese Medicine and used for treating inflammation and cancer in clinical formulas. Neobaicalein (NEO) is a kind of flavonoid (Figure 1) isolated from Huangqin and has been reported to possess prominent anti-inflammatory effects. Current research revealed its neuroprotective effects on rotenone-induced cell toxicity and LPS-induced inflammation [17] and promoted cell apoptosis in Hela cells [18], HepG2 and MCF-7 cells [19]. However, the influence of NEO on diverse autoimmune inflammatory diseases has not been reported yet. In the present study, we initially discovered the involved predominant mechanisms for NEO on IL-17 signaling pathway through network pharmacology-based study. Based on that IL-17 is unique cytokines secreted by Th17 cells, influence of STAT3 signaling on cell proliferation, Th17 differentiation and RORγt expression [14,15] and we found NEO effectively inhibited phosphorylation of STAT3(Tyr725) stimulated by IL-6 in splenocytes. We then suspected whether NEO suppressed Th17 cell differentiation and was to be a potential candidate for treating autoimmune inflammatory diseases. Therefore, we put forward the hypothesis of whether NEO effectively interferes Th17 cell differentiation through blocking STAT3 signaling. Thus, effect of NEO on differentiation of CD4^+^ T cells to Th17 cells and its influence on STAT3 signaling was investigated in the present study.

## 2. Results

### 2.1. IL-17 Signaling Pathway Regulated by NEO Was Discovered Both RA, IBD and MS

**Figure 2:**
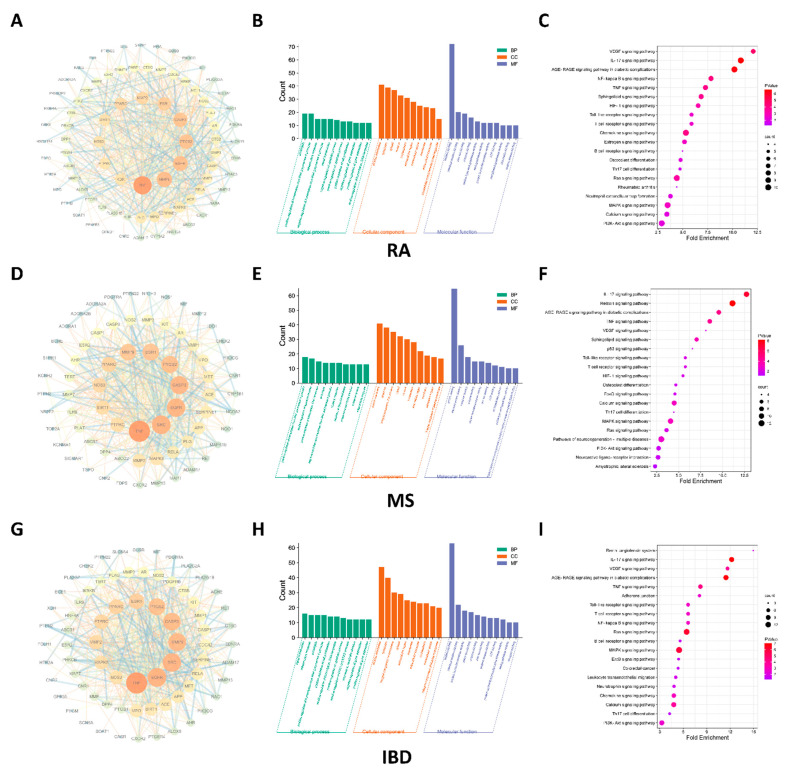
PPI network regarding shared targets of NEO and RA (**A**), MS (**D**) and IBD (**G**); GO analysis of shared targets of NEO and RA (**B**), MS (**E**) and IBD (**H**); KEGG analysis for signaling pathway of shared targets of NEO and RA (**C**), MS (**F**) and IBD (**I**).

PPI network analysis revealed that 84 genes were influenced by NEO in RA, 74 genes in IBD and 73 genes in MS (Figure 2A,D,G). Moreover, GO analysis demonstrated that signal transduction and inflammatory response at biological process levels, and plasma membrane at cellular component levels and protein binding at molecular function levels (Figure 2B,E,H). Additionally, IL-17 signaling pathway regulated by NEO was discovered as the top cascade in the three diseases by assessing fold enrichment and count (Figure 2C,F,I).

### 2.2. NEO Significantly Inhibited ConA-Stimulated CD4^+^ T Proliferation

**Figure 3:**
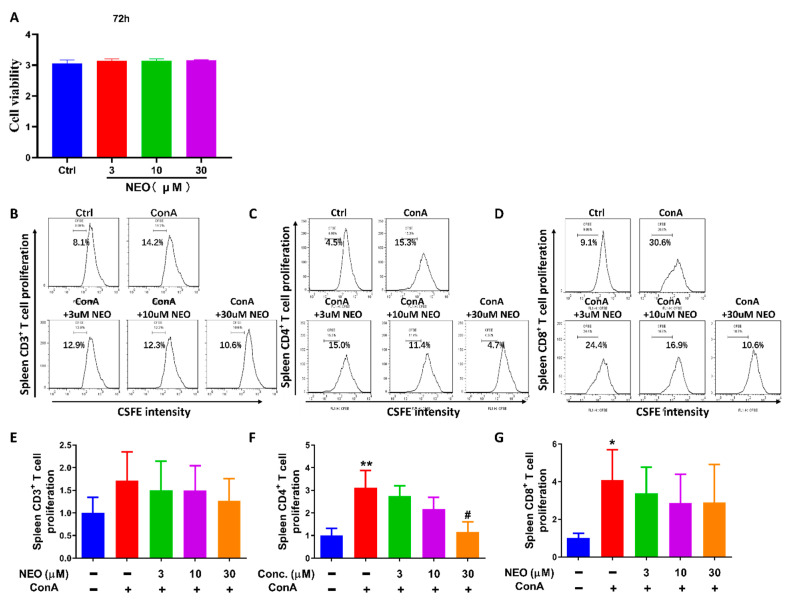
Effect of NEO on splenocytes viability and T cell proliferation. (**A**) Impact of NEO on splenocytes viability. (**B**,**E**) Effect of NEO on CD3^+^ T cell proliferation. (**C**,**F**) Inhibition of NEO on CD4^+^ T cell proliferation. (**D**,**G**) Effect of NEO on CD8^+^ T cell proliferation. The results represent three independent experiments. * *p* < 0.05, and ** *p* < 0.01 indicated that the differences were statistically significant vs. control group; whereas, ^#^ *p* < 0.05 declared statistically significant differences vs. ConA-stimulated group (n = 3).

Based on result regarding network pharmacology-based study, IL-17 signaling pathway was predominantly regulated by NEO. Moreover, IL-17 is unique cytokines secreted by Th17 cells. We further investigated influence of NEO on Th17 cells. In order to investigate the effect of NEO on splenocytes and confirm the safety of NEO, CCK8 assay was utilized here to exclude toxic impacts of NEO. Viewed from Figure 3A, NEO failed to inhibit cell viability at 30 μM, which was the highest dose in the experiment. Then, we further analyzed weather NEO suppressed concanavalin A (ConA)-stimulated splenocytes proliferation; thus, the CFSE assay were applied to evaluate the proliferation of CD3^+^, CD4^+^ and CD8^+^ splenocytes. Results showed that ConA significantly increased CD4^+^ and CD8^+^ cell proliferation when compared with unstimulated control cells. NEO significantly blocked ConA-stimulated CD4^+^ splenocytes proliferation at the dose of 30 μM, whereas NEO treatment slightly inhibited CD3^+^ and CD8^+^ cell proliferation and this reduction did not reach statistically significance.

### 2.3. NEO Blocked CD4^+^ T Activation through Decreasing CD25 and CD69 Expression

**Figure 4:**
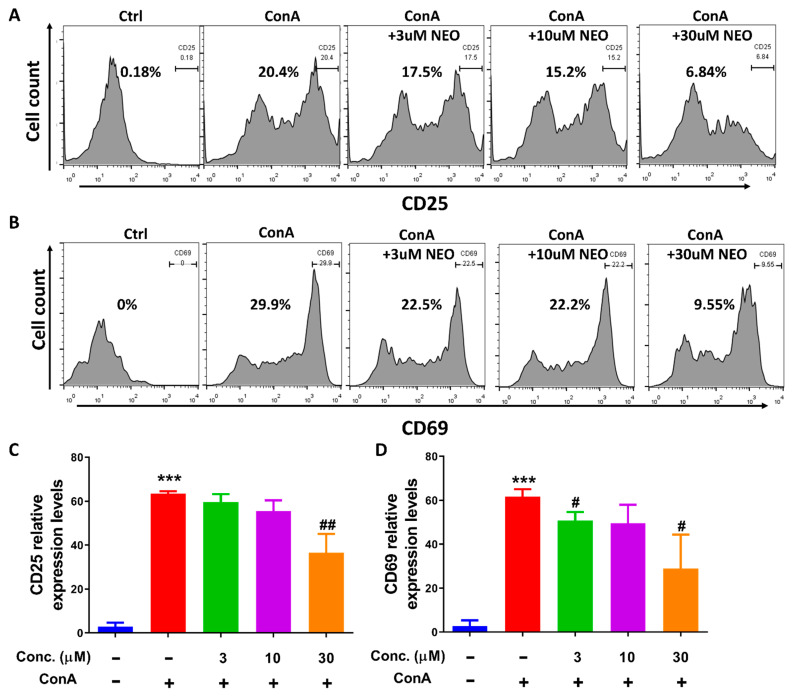
Effect of NEO on CD4^+^ T cell activation. (**A**,**C**) NEO suppressed CD25 expression stimulated by ConA. (**B**,**D**) NEO suppressed CD69 expression stimulated by ConA. The results represent three independent experiments. *** *p* < 0.001 indicated that the differences were statistically significant vs. control group; whereas, ^#^ *p* < 0.05, and ^##^ *p* < 0.01 declared statistically significant differences vs. ConA-stimulated group (n = 3).

T cell activation is essential to subsequent T cell proliferation, CD25 and CD69 are markers of T cell initial activation in response to specific signal or non-specific signal stimulation. Therefore, we investigated effect of NEO on CD25 and CD69 expression stimulated by ConA, which activated T cells non-specifically. Results showed that both CD25 and CD69 in CD4^+^ T cells were increased up to approximately 100 folds compared to control group (Figure 4); however, NEO significantly reversed high levels of CD25 and CD69 at the dose of 30 μM.

### 2.4. NEO Restricted the Differentiation of CD4^+^ T Cells to Th17 Cells

**Figure 5:**
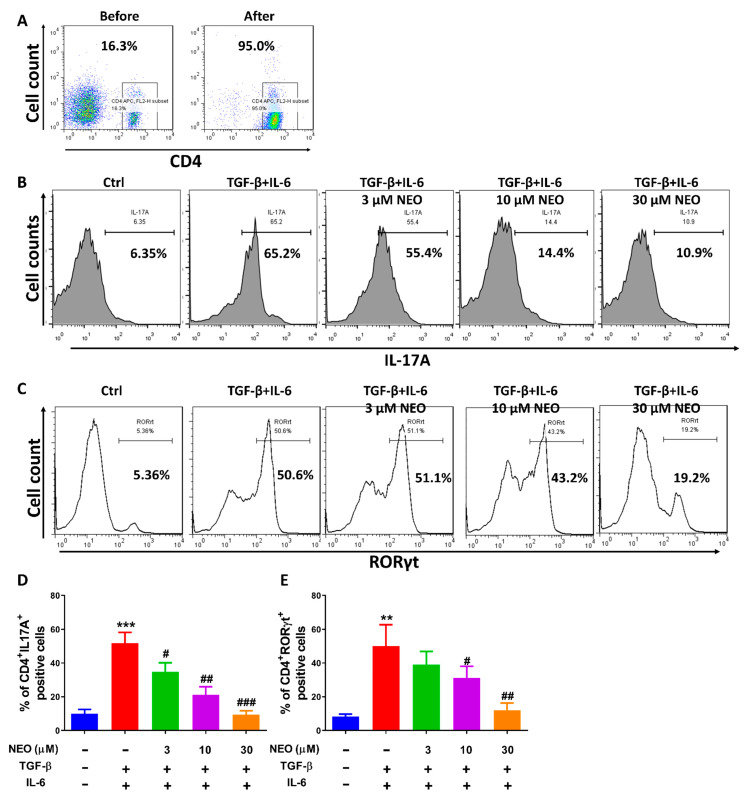
NEO restricted the differentiation of CD4^+^ T cells to Th17 cells. (**A**) Purity of CD4^+^ T cell before and after magnetic separation. (**B**,**D**) Effect of NEO on the percentage of population of CD4^+^IL-17A^+^ T cells. (**C**,**E**) Effect of NEO on the percentage of population of CD4^+^RORγt^+^ T cells. The results represent three independent experiments. ** *p* < 0.01 and *** *p* < 0.001 indicated that the differences were statistically significant vs. control group; whereas, ^#^ *p* < 0.05, ^##^ *p* < 0.01 and ^###^ *p* < 0.001 declared statistically significant differences vs. TGF-β and IL-6 group (n = 3).

Th17 cells, subtype of CD4^+^ T cells, have been reported to accelerate autoimmune and infected diseases pathogenesis [20]. In this regard, we reasonably suspected that there was a possibility of NEO inhibiting Th17 differentiation. Thus, we performed CD4^+^ T cells sorting by negative magnetic separation technology for the purified CD4^+^ T cells and the subsequent differentiation of CD4^+^ T cells to Th17 cells. First, our results revealed that the purified CD4^+^ T cells were obtained successfully, whose purity was up to 95% (Figure 5A, right). Second, the purified CD4^+^ T cells were induced to differentiate to Th17 cells in the presence of TGF-β and IL-6, which was established to investigate the effect of NEO on Th17 differentiation. Results demonstrated that percentage of Th17 differentiation in TGF-β and IL-6 stimulated group reached up to 65.8%, whereas less Th17 cells (4.33%) were detected in control group. Interestingly, NEO could still reverse the intensive increase in Th17 cells, which manifested as decrease in CD4^+^IL-17A^+^ cell population (Figure 5B,D) and CD4^+^RORγt^+^ cell population (Figure 5C,E).

### 2.5. NEO Extinguished Cytokine Secretion in the Process of Differentiation of CD4^+^ T Cells to Th17 Cells

**Figure 6:**
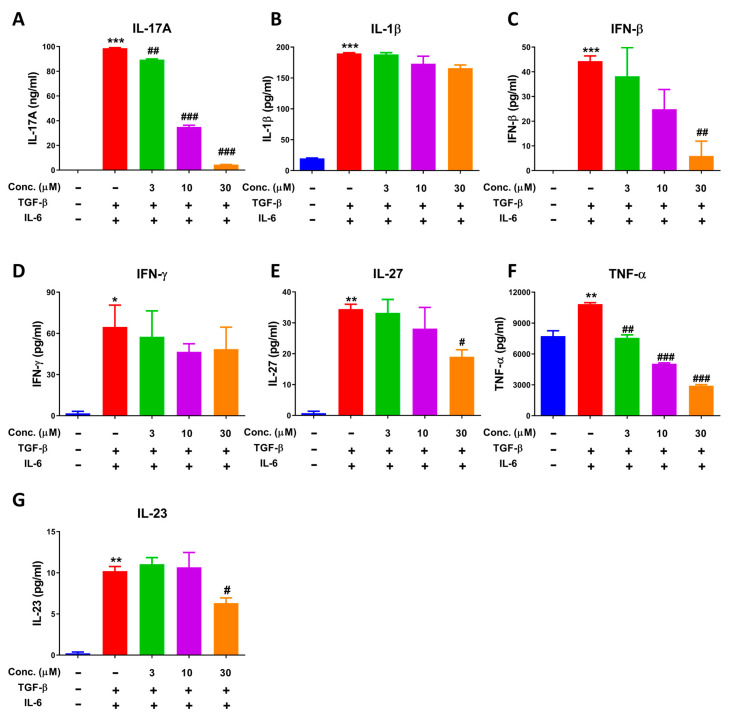
Effect of NEO on cytokines in the process of Th17 cell differentiation. (**A**–**G**) Impacts of NEO on individual cytokines levels. * *p* < 0.05, ** *p* < 0.01 and *** *p* < 0.001 indicated that the differences were statistically significant vs. control group; whereas, ^#^ *p* < 0.05, ^##^ *p* < 0.01 and ^###^ *p* < 0.001 declared statistically significant differences vs. TGF-β and IL-6 group (n = 3).

Various cytokines were produced when CD4^+^ T cells differentiated to Th17 cells; therefore, we analyzed the levels of cytokines by CBA assay. Results regarding profile of cytokines in Th17 differentiation showed that amounts of IL-17A were produced and reached up to nearly 10,000-fold compared to control group, which was the most significantly increased compared to other cytokines (Figure 6A–G). Additionally, as illustrated in Figure 6A,C,F, IL-17A, IFN-β and TNF-α was significantly inhibited by NEO in a dose-dependent manner. Additionally, IL-1β, IL-23 and IL-27 was decreased significantly at high dose of 30 μM. However, IFN-γ was only observed a slight tendency towards inhibition, but no significance was recorded.

### 2.6. NEO at a High Dose Reduced Treg Cell Differentiation

**Figure 7:**
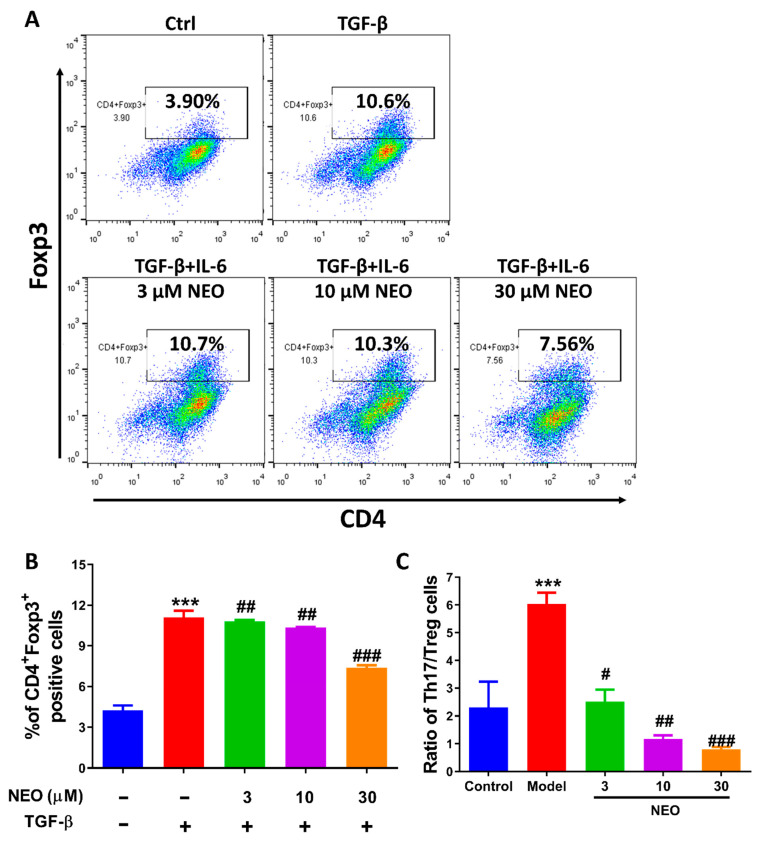
Influence of NEO on Treg cell differentiation. (**A**,**B**) Effect of NEO on CD4^+^ Foxp3^+^ cell population. (**C**) Recovery ratio of Th17/Treg cells caused by NEO. The results represent three independent experiments. *** *p* < 0.001 indicated that the differences were statistically significant vs. control group; whereas, ^#^ *p* < 0.05, ^##^ *p* < 0.01 and ^###^ *p* < 0.001 declared statistically significant differences vs. TGF-β group (n = 3).

The balance between Th17 and Treg cells are crucial factor in maintaining inflammation and immune tolerance [21]. Thus, we also analyzed the effect of NEO on Treg cells. Results showed that NEO almost had no inhibitory effect on CD4^+^Foxp3^+^ cells at the dose of 3 μM and 10 μM. However, a slight decrease in CD4^+^Foxp3^+^ cells were observed at a high dose of NEO (Figure 7A,B). The ratio of Th17/Treg cells was significantly down-regulated as calculated from the obtained data (Figure 7C).

### 2.7. NEO Inhibited Phosphorylation of STAT3 and STAT3 Nucleus Translocation

**Figure 8:**
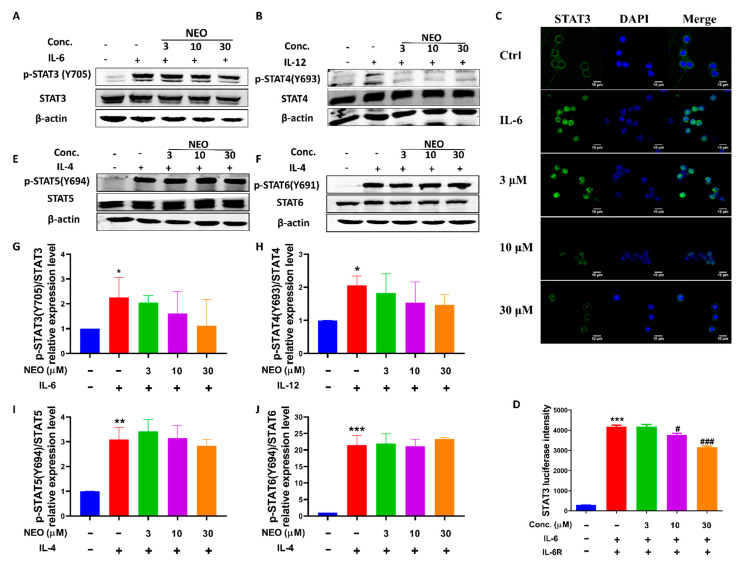
Blockage in STAT3 signaling by NEO. Effects of NEO on the levels of p-STAT3(Tyr725) (**A**,**G**), p-STAT4 (Y693) (**B**,**H**), p-STAT5 (Y694) (**E**,**I**), p-STAT6 (Y641) (**F**,**J**) in splenocytes stimulated with the indicated cytokines. The results represent three independent experiments. (**C**) NEO restricted nucleus location of STAT3. (**D**) NEO down-regulated STAT3 transcriptional activity. * *p* < 0.05, ** *p* < 0.01 and *** *p* < 0.001 indicated that the differences were statistically significant vs. control group; whereas, ^#^ *p* < 0.05, and ^###^ *p* < 0.001 declared statistically significant differences vs. IL-6, IL-12 or IL-4 group (n = 3).

Amounts of evidence reported that STAT3 is another monitor regulating both RORγt expression and cytokines in the process of Th17 differentiation [14]. In this regard, the effect of NEO on STAT3 signaling activation was evaluated. Western blotting results revealed that NEO had tendency to restrain phosphorylation of STAT3 in every independent experiment, but no significance was observed in the three independent experiments because of the big standard deviation (Figure 8A,G). Additionally, STAT3 is transcription factor and the function of activated STAT3 relies on its translocation from cytoplasm into the nucleus. Thus, we detected whether NEO inhibited STAT3 translocation into nucleus. Immunocytochemistry assay results demonstrated that IL-6 facilitated STAT3 translocation into nucleus; however, NEO could inhibit STAT3 translocation into nucleus (Figure 8C). The luciferase reporter assay also revealed that NEO could reverse high STAT3 luciferase levels in the presence of IL-6 and IL-6R after transfected with pSTAT3-TA-luc and pRL-SV40 (Figure 8D). Interestingly, NEO also had a slight tendency to inhibit phosphorylation of STAT4 stimulated by IL-12 (Figure 8B,H) and performed no influences on phosphorylation of STAT5 and STAT6 induced by IL-4 (Figure 8E,F,I,J).

### 2.8. ADME Properties and Drug-Likeness of NEO

*p*-values were used to evaluate the solubility properties of a compound in octanol and water and the results show that NEO has good drug-likeness properties, as shown in Table 1. The pharmacokinetic property presented in Table 1 revealed that NEO show a good performance of absorption and it did not affect CYP2C19 and CYP2D6, but was evaluated as inhibitor of CYP1A2 and CYP2C9, which is a vital enzyme of drug metabolism. Additionally, NEO did not cross the blood-brain barrier and was not P-gp substrate, which was assisted intracellular drug delivery (Table 1).

## 3. Discussion

Autoimmune diseases are widespread in clinics all over the world, including rheumatoid arthritis (RA), multiple sclerosis (MS), inflammatory bowel disease (IBD), psoriasis and so on [1,2]. However, the current therapies fail to meet the requirements for treatment of RA, MS and IBD. Therefore, it is urgent to discover new therapeutic drugs.

The Th17/Treg balance shift to Th17 cells is the most critical cause for autoimmune inflammatory diseases and the differentiation of Th17 and Treg cells is determined by activation of signal transducer and activator of transcription 3 (STAT3). Neobaicalein (NEO) is a kind of flavonoid (Figure 1) isolated from *Scutellaria baicalensis* Georgi. Network pharmacology-based study revealed that NEO predominantly regulated IL-17 signaling pathway and IL-17 is a unique cytokine secreted by Th17 cells. The present study reported NEO inhibited Th17 cell differentiation, but had negative inhibitory effect on Treg cells, which resulted in decreased ratio of Th17/Treg together with Th17 related cytokines, such as IL-17A and TNFα. Based on influence of STAT3 signaling on cell proliferation, Th17 differentiation and RORγt expression [14,15], we determined effect of NEO on differentiation of CD4^+^ T cells to Th17 cells and its influence on STAT3 signaling in the present study. Our results demonstrated that NEO (3–30 μM) could dose-dependently inhibit Th17 cell differentiation as evidenced by the decreasing proportion of CD4^+^IL-17A^+^ cells and CD4^+^RORγt^+^ cells, which was a symbol of successfully Th17 cell differentiation. Accordingly, NEO effectively reduced IL-17A and TNFα release, two important proinflammatory cytokines produced by Th17 lymphocytes, in a dose-dependent manner during Th17 cell differentiation as assessed by CBA assay. These effects were not attributed to its cytotoxicity for the reason that cell cytotoxicity was not observed at concentrations below 30 μM. Moreover, the further mechanism research revealed that NEO could markedly inhibit phosphorylation of STAT3(Tyr725), effectively blocked STAT3 translocated into nucleus, and considerably reduced STAT3 transcriptional activity. These results suggest that NEO effectively interferes Th17 cell differentiation through blocking STAT3 signaling.

T cells activation is essential to the subsequent cell proliferation, differentiation and cytokine release. Using ConA-stimulated splenocytes as an in vitro model, we found that only at high concentration, NEO extinguished the cell surface expression of CD25 and CD69, the marker of T cell activation, and significantly reduced proliferation of CD4^+^ T cells in splenocytes. These effects were different from that NEO in a dose-dependent manner blocked Th17 differentiation and corresponding cytokine production, which indicates that NEO suppressed Th17 differentiation independently of T lymphocyte activation and proliferation. Additionally, our data showed that NEO failed to inhibit Treg differentiation at doses of 3 μM and 10 μM, respectively. In contrast, a high dose of NEO at 30 μM resulted in less Treg differentiation. Moreover, NEO had not influence on activation of STAT5, which activates transcription of Foxp3 and influence Treg differentiation and survival [22]. These results suggest that inhibitory effect of NEO (30 μM) on Treg differentiation may be attributed to its inhibitory influences on T-cell activation and proliferation. Overall, Th17 cells were the major interference cells by NEO through inhibiting STAT3 signaling. Excessive function of Th1 cells, which produce interferon-γ (IFN-γ) is generally associated with various autoimmune disorders [23]. STAT4 has been reported to be associated with Th1 differentiation. Our results found that NEO inhibited activation of STAT3 and STAT4 signaling without suppressing phosphorylation of STAT5 and STAT6, suggesting that NEO possibly had double antagonized effects on both Th1 and Th17 cell differentiation.

## 4. Materials and Methods

### 4.1. Reagents

Total spleen cells were isolated from spleen of C57B/L6 male mice [24], naïve CD4^+^ T cells and CD4^+^ T cells were purified from splenocytes through magnetic separation technology. C57B/L6 mice (male, 20–22 g, 8 weeks) were purchased from Shanghai Jiagan Biotechnology Co., Ltd. (Shanghai, China); NEO (>97.7% purity verified by HPLC) was purchased from SiChuan RuiFenSi Medical Technology Co. (SiChuan, China). Naïve CD4^+^ T cells isolation kits were purchased from Miltenyi Biotec (Bergisch Gladbach, Germany). Primary antibodies, including p-STAT3(Tyr705), p-STAT4(Y693), p-STAT5(Y694), p-STAT6(Y641) and STAT3, STAT4, STAT5 and STAT6 were purchased from Cell Signaling Technology (Boston, USA); β-actin was obtained from Santa Cruz Biotechnology (Santa Cruz, CA, USA). CD3, CD4, CD8, CD25, CD69, Foxp3, RORγt, IL-17A antibody and CFSE label-probe were obtained from BD Biosciences (San Jose, CA, USA). Lipofectamine 3000 kits were provided by Invitrogen (Carlsbad, CA, USA); RPMI 1640 Medium, OPTI-Minimum Essential Medium (MEM), Fetal bovine serum (FBS), Fetal bovine serum (FBS) and 100 × penicillin-streptomycin (P/S) were purchased from Gibco (Paisley, UK). Phosphate Buffered Saline (PBS) were obtained from Invitrogen (Carlsbad, CA, USA). Cholecystokinin octapeptide (CCK8) and Dimethylsulfoxide (DMSO) were purchased from Sigma Aldrich (St. Louis, MI, USA).

### 4.2. Network Pharmacology-Based Study

Targets involved in RA, IBD and MS pathogenesis were collected by OMIM, GeneCards and TTD database. Additionally, intersection of predicted targets of NEO and RA, IBD and MS were screened by venn website. Subsequently, STRING database and Cytoscape3.8.2 software was used to draw PPI targets in RA, IBD and MS pathogenesis. Moreover, GO and KEGG enrichment was analyzed to get corresponding information regarding effect of NEO on biological function (BP), molecular function (MF) and cell components (CC) and signaling pathway [25].

### 4.3. CCK8 Assay

Splenocytes were isolated from spleens of healthy male C57B/L6 mice and spleen cells (1 × 10^6^/well) were seeded into 96 well plates. To verify whether NEO inhibits splenocytes viability or not, splenocytes were treated with NEO for 72 h; afterward, CCK8 reagent was added into each well and incubated with cells for 4 h. Finally, OD values were recorded by OD plate reader (Infinite M200 PRO, Germany) at λ = 450 nm [26].

### 4.4. Splenocytes Proliferation Assay

To evaluate the effect of NEO on the proliferation of splenocytes, splenocytes were pre-stained with CFSE (5 μM) for 10 min. After that, the stained cells were washed with PBS twice and seeded into 24-well plates. Then, cells were pretreated with NEO for 4 h and co-incubated with ConA (5 μg/mL) for another 72 h. Subsequently, splenocytes were collected and rinsed with PBS and stained with CD3^+^, CD4^+^ and CD8^+^ antibody for 30 min, respectively. Finally, cells were collected and detected by Fluorescence Activating Cell Sorter (FACS) [27].

### 4.5. Splenocytes Activation Assay

To detect the effect of NEO on the activation of splenocytes, splenocytes were seeded into 24-well plates. Then, cells were pretreated with NEO for 4 h and co-incubated with ConA (5 μg/mL) for another 12 h. Subsequently, splenocytes were collected and rinsed with PBS and stained with CD4^+^ and CD25^+^ or CD69^+^ antibody for 30 min, respectively. Finally, cells were collected and detected by FACS [28].

### 4.6. T Cells Differentiation Assay

Naïve CD4^+^ T cells were isolated from splenocytes by negative selection kit (Miltenyi Biotec, Auburn) and cell purity (>95%) were monitored by Flow cytometry. The purified naïve CD4^+^ T cells were plated into 24-well plates, were treated with NEO for 4 h in the presence of CD3 (5 μg/mL) and CD28 (1 μg/mL). To verify whether NEO inhibits Th17 cells differentiation, cells were followed by TGF-β (2.5 ng/mL) and IL-6 (30 ng/mL) stimulation for another 72 h. To verify whether NEO inhibit Treg cells differentiation, cells were followed by TGF-β (5 ng/mL) stimulation for another 72 h. Subsequently, cells were collected and fixed for 1 h, permed twice and each time for 15 min. Afterward, cells were stained with CD4, RORγt and IL-17A antibody for Th17 differentiation assay and stained with CD4, CD25 and Foxp3 antibody for Treg differentiation assay, respectively. Finally, the stained cells were analyzed by FACS.

### 4.7. Cytometric Bead Array (CBA) Assay

The purified naïve CD4^+^ T cells were plated into 24-well plates and were treated with NEO for 4 h in the presence of CD3 (5 μg/mL) and CD28 (1 μg/mL) and followed by TGF-β (2.5 ng/mL) and IL-6 (30 ng/mL) stimulation for another 72 h. Afterward, supernatants were collected and CBA assay were performed for evaluating the levels of cytokines. CBA assay refers to the protocol of product manual.

### 4.8. Western Blot

To investigate whether the expression of STAT3, STAT4, STAT5 and STAT6 was regulated by NEO, splenocytes were plated into the 12-well plates and treated with NEO for 1 h and then incubated with IL-6, IL-12 or IL-4 for another 30 min. The total cellular protein was extracted and western blotting according to our previous work [28].

### 4.9. Immunocytochemistry

To verify whether NEO induced the nuclear translocation of STAT3, immunocytochemistry was performed on splenocytes incubated with NEO. To be specific, splenocytes were seeded onto the coverslips in the 6-well plates and incubated overnight, followed by 1 h of NEO treatments. Additionally, the immunofluorescence cell staining was conducted according to our previous report [28].

### 4.10. Luciferase Reporter Assay

HEK293T cells (1 × 10^7^/dish) were transfected with 27 μg pSTAT3-TA-luc and 9 μg pRL-SV40 by lipo3000. After 6 h, cells were collected by trypsin and plated into 96-well plates (1 × 10^4^/well), followed by treatment with NEO for 24 h, then, cells were co-incubated with IL-6 and IL-6R for another 24 h. Finally, cells were collected and luciferase assay were performed, which refers to our previous work [28].

### 4.11. ADME and BBB Assay

Absorption, distribution, metabolism and excretion (ADME) and blood brain barrier (BBB) prediction of NEO was analyzed through Swiss ADME server and online BBB prediction tool [29,30].

### 4.12. Statistical Analysis

Data were presented as means ± SEM. One-way ANOVA with the Tukey’s post-hoc test in GraphPad Prism was adopted to analyze the significance of differences. * *p* < 0.05, ** *p* < 0.01 and *** *p* < 0.001 indicated that the differences were statistically significant vs. control group; whereas, ^#^ *p* < 0.05, ^##^ *p* < 0.01 and ^###^ *p* < 0.001 declared statistically significant differences vs ConA, TGF-β and IL-6 group.

## 5. Conclusions

The present study revealed that NEO effectively blocked Th17 differentiation and production of corresponding cytokines in the process of Th17 differentiation, which is possibly attributed to specifically inhibitory effects on STAT3 signaling. Down-regulation of Th17/Treg cell ratio by NEO may be important in antagonizing autoimmune inflammatory diseases. NEO was not P-gp substrate and had good properties of intracellular drug delivery, whereas NEO did not cross the blood-brain barrier, suggesting that it was more effective to antagonize inflammation in periphery than that in CNS. Our work provided support that NEO was to be a potential candidate for treating autoimmune inflammatory diseases. However, in vivo experiment regarding arthritis or IBD should be performed in the future to confirm its effect on autoimmune inflammatory diseases.

## Figures and Tables

**Table 1:** ADME properties and drug-likeness of NEO.

Properties	Parameters	Neobaicalein
Physicochemical Properties	MW^a^ (g/mol)	374.34
	Rotatable bonds	5
	H-Bond Acceptors	8
	H-Bond Donors	2
	Fraction Csp3	0.21
	TPSA	107.59 Å²
Lipophilicity Log *P*o/w	iLOGP	3.11
	XLOGP3	2.91
	MLOGP	−0.12
	Consensus	2.4
Absorption	Water solubility	−4.10
	GI absorption	High
	Log *K*p (skin permeation) cm/s	−6.52
Metabolism	CYP1A2 inhibitor	Yes
	CYP2C9 inhibitor	Yes
	CYP2C19 inhibitor	No
	CYP3A4 inhibitor	Yes
	CYP2D6 inhibitor	No
	BBB	No
	P-gp substrate	No
Drug-likeness	Lipinski	Yes
	Ghose	Yes
	Veber	Yes
	Egan	Yes
	Muegge	Yes
	Bioavailability score	0.55

## Data Availability

Not Applicable.

## Abbreviations

DMSO	Dimethylsulfoxide
FACS	Fluorescence Activating Cell Sorter
IBD	Inflammatory Bowel Disease
MMPs	Matrix Metalloproteinases
MS	Multiple Sclerosis
NEO	Neobaicalein
PBS	Phosphate Buffered Saline
RA	Rheumatoid Arthritis
RORγt	Retinoic acid receptor-related orphan nuclear receptor
STAT3	Signal Transducer and Activator of Transcription 3
Th17	T helper 17
Treg	Regulatory T cells
